# Seeing Through Other Eyes: How Language Experience and Cognitive Abilities Shape Theory of Mind

**DOI:** 10.3390/bs15060755

**Published:** 2025-05-30

**Authors:** Manali Pathare, Ester Navarro, Andrew R. A. Conway

**Affiliations:** 1 Department of Psychology, New Mexico State University, Las Cruces, NM 88003, USA; aconway@nmsu.edu; 2 Department of Psychology, St. John’s University, Queens, NY 11439, USA

**Keywords:** theory of mind, bilingualism, social cognition, individual differences

## Abstract

Understanding others’ perspectives, or Theory of Mind (ToM), is a critical cognitive skill essential for social competence and effective interpersonal interactions. Although ToM is present in varying degrees across individuals, recent research indicates that linguistic factors, particularly bilingualism, can significantly influence its expression. Building on these findings, the current study examined performance on the perspective-taking trials of the Director Task among bilinguals and monolinguals. The results showed a nonsignificant trend in accurate responses as a function of bilingualism; however, a significant effect was found when examining only perspective-taking trials, with bilinguals outperforming monolinguals, suggesting that larger sample sizes are needed to identify this effect. Interestingly, a significant interaction between fluid intelligence and bilingualism was found, suggesting that bilinguals with higher fluid intelligence performed better on perspective-taking trials compared to bilinguals with lower fluid intelligence. The results emphasize the importance of domain-general abilities for the effect of bilingualism on perspective-taking and suggest that bilingualism’s effect on ToM may be more salient in individuals with higher cognitive abilities.

## 1. Introduction

Effective navigation of complex social interactions requires individuals to recognize that the behavior of others is influenced by mental states that are not directly observable—such as beliefs, desires, intentions, and emotions. This foundational ability, known as Theory of Mind (ToM) or mentalizing, plays a critical role in social competence, communication, and conflict resolution (Sperber & Wilson, 1995). ToM allows individuals to understand perspectives and ideas that differ from their own (Perner, 1988; Wellman, 1990), and its development is considered a major milestone in early cognitive and social development. In addition, ToM abilities have been linked to stronger problem-solving skills, executive functioning, and reasoning about conflicting perspectives (German & Hehman, 2006; Greenberg et al., 2013). In fact, deficits in ToM are frequently observed in individuals with social-cognitive impairments, such as autism spectrum disorder (Baron-Cohen et al., 1985) and other neuro-developmental conditions (Hughes & Russell, 1993).

Although ToM is typically associated with childhood development, research increasingly highlights its relevance and variability in adulthood. Adults often struggle with ToM-relevant tasks, especially in conditions of high cognitive load, time pressure and stress (Navarro et al., 2020), and in situations where their own knowledge must be inhibited to accurately infer another’s perspective (Keysar et al., 2003; Birch & Bloom, 2007). This egocentric bias can lead to communication breakdowns and social misunderstandings (Mitchell et al., 2009). A growing body of work suggests that experience-based factors, such as language exposure, may shape individual differences in ToM performance. Bilingualism—the knowledge of two languages (Valdez & Figueora, 1994)—has been proposed as one such factor. Early research with children identified performance differences between bilinguals and monolinguals in false belief tasks (Goetz, 2003). This research suggested that bilingual individuals, who must consciously identify and switch between language systems based on the language of their interlocutor using controlled mechanisms, may be able to extrapolate this ability to identify and switch between the mental states and perspectives of their interlocutor more broadly (Kloo & Perner, 2003). While the exact mechanisms underlying these observed effects are not yet clear, researchers have suggested that bilingualism influences mental state attribution (Goetz, 2003) due to the increased need to inhibit, switch, or update one language’s rules (e.g., syntax, morphology, phonology) and pragmatic cues (e.g., common ground, turn-taking, implied meaning) from an early age (Genesee et al., 1996) and often in rapid on-line social interactions (Green & Abutalebi, 2013). Interestingly, while early research assumed that adult ToM, in the absence of deficits, was stable and constant (Keysar et al., 2003), subsequent findings have shown that ToM is a skill that fluctuates throughout the life span (Dumontheil et al., 2010; Raimo et al., 2022; Goring & Navarro, in press).

Along similar lines, various cognitive abilities have been shown to influence ToM, such as Fluid intelligence (Gf). Gf broadly defined, is our capacity to reason through novel problems independent of acquired knowledge (Cattell, 1963), and it supports cognitive flexibility, abstraction, and problem-solving that is often required in ToM tasks (I. A. Apperly et al., 2010; German & Hehman, 2006; Navarro, 2022). For instance, psychosocial adaptation and social context have been linked to Gf, suggestive of its relevance to social cognition (Huepe et al., 2011). Additionally, Gf has been associated with facial processing and the Reading the Mind in the Eyes Test (RMET) (Wilhelm et al., 2010; Roca et al., 2010), alluding to its importance in emotion recognition, an important aspect of ToM (Mier et al., 2010). To contribute to the growing literature, the present study expands upon the work of Navarro and Conway (2021) by examining individual differences in adult ToM performance among bilinguals and monolinguals. Specifically, we investigate whether bilingualism is associated with enhanced performance on perspective-taking ToM tasks, and whether this relationship is moderated by fluid intelligence. While other domain-general abilities such as working memory or attention control have also been linked to ToM performance, we focus on Gf due to its central role in abstract reasoning, inhibition, and novel problem solving—all of which are critical in managing conflicting perspectives (German & Hehman, 2006; Roca et al., 2010). By taking a multivariate, individual-differences approach, our study seeks to narrow the mechanisms through which bilingual experience—and its interaction with cognitive abilities—may shape ToM abilities in adulthood.

## 2. Contemporary Research on Adult Theory of Mind

Theory of mind (ToM) is closely tied to several fundamental cognitive abilities, such as vocabulary and executive control (Astington & Baird, 2005; German & Hehman, 2006; Carlson & Moses, 2001). As such, research has long indicated that developing core cognitive abilities is a requisite to completing ToM tasks, especially during highly taxing ToM situations (e.g., conflicting perspectives). Thus, the need to develop and successfully engage domain-general abilities to complete ToM tasks indicates that ToM can be subject to individual differences.

In fact, extensive research has shown that performance is influenced by task demands, mental resource availability, and inhibitory mechanisms. For example, adults tend to make egocentric (i.e., failure to inhibit one’s own thoughts, perspectives, or knowledge) errors when completing tasks that require them to hold other perspectives in working memory, inhibit one’s own knowledge or perspective, or ascribe mental states to others (Keysar et al., 2003; I. A. Apperly et al., 2008; I. A. Apperly & Butterfill, 2009; Birch & Bloom, 2007; Mitchell et al., 2009; Navarro et al., 2020). Yet, little research has explored the mechanisms underlying this variability.

One of the first studies conducted to identify variability in ToM performance across development was conducted by Dumontheil et al. (2010). The researchers found that performance in the Director Task of perspective-taking (Keysar et al., 2003) did not stabilize after childhood; instead, performance improved steadily as a function of age, with young adults outperforming adolescents, who in turn outperformed children. The age-related improvements in perspective conflict resolution were attributed to the maturation of ToM and executive function interactions, supporting neuroimaging research showing continuous development of areas involved in mental state attribution (i.e., medial prefrontal cortex and lateral temporo-parietal regions) that continue to develop during adolescence at the structural and functional levels (Giedd et al., 1999; Shaw et al., 2008; Blakemore, 2008). Critically, the authors proposed that the improvements observed in the perspective-taking condition (but not control conditions) between adolescents and adults suggested that conflicting perspective-taking resolution continues to improve even after adolescence. In contrast, participants’ general working memory and inhibitory task demands, which are unrelated to the perspective conflict condition, demonstrated adult-level ability.

The findings that perspective conflict resolution varies across individuals after accounting for domain-general ability suggests that performance variability at the individual level may be influenced by external factors. For example, individual differences in early ToM development in children have been associated with variability in language ability, executive function, and social environment (Milligan et al., 2007; Devine & Hughes, 2014), emphasizing the importance of experience-based modulation on performance. In fact, language ability, and more specifically syntactic complexity and vocabulary, facilitate early expression and comprehension of mental states (Milligan et al., 2007). Similarly, individual differences in performance among older adults has been associated with declines in cognitive reasoning and problem-solving as a result of healthy aging, while tasks that require social expertise and emotion ascription seem to present different trajectories (Henry et al., 2013; Grainger et al., 2023). Overall, these findings suggests that cognitive changes across the lifespan play an important role in changes in ToM, but the impact of these changes on performance may fluctuate based on experience-related factors like social competence, linguistic ability, and emotional processing (Goring & Navarro, in press). Given the benefits of maintaining a healthy ToM throughout the lifespan for interpersonal functioning (Bailey & Henry, 2008; Yeh, 2013), well-being and successful aging (Carstensen et al., 2003; Henry et al., 2013), it is crucial to examine how environmental factors influence ToM—whether as a function of, or independent from, domain-general abilities.

### 2.1. The Relationship Between Bilingualism and Theory of Mind

Numerous studies have reported differences in performance between monolingual and bilingual children (Goetz, 2003; Kovács, 2009; Greenberg et al., 2013; Diaz & Farrar, 2017, for a meta-analysis, see Schroeder, 2018). This research suggests that an interplay of general cognitive abilities, sociocultural exposure, family context (e.g., having siblings) and metalinguistic skills may underlie these findings (Wu & Keysar, 2007; Cutting & Dunn, 1999; Wellman, 2018). Specifically, some researchers suggest that bilinguals may have enhanced attention control because of the constant switch between, and inhibition of, both of their languages, which in turn enhances domain-general cognitive abilities (e.g., Bialystok, 2009; Bialystok & Craik, 2010). According to this view, enhanced attentional control may support bilinguals’ advantage in ToM tasks by facilitating the inhibition of their own perspective in favor of another’s (Bialystok & Senman, 2004; Carlson & Moses, 2001; A. Leslie, 1994; A. M. Leslie et al., 2005; Scholl & Leslie, 2001). However, until recently it was not clear whether these group differences would be observed among older individuals. In particular, young adults are an interesting population because researchers generally do not find consistent evidence of differences in performance of executive function tasks among them (Nichols et al., 2020), which has been attributed to young adults exhibiting peak cognitive ability. Therefore, differences in ToM among these groups could be largely related to other factors.

For example, Rubio-Fernandez and Glucksberg (2012) found that college-aged bilinguals presented fewer false belief detection errors, measured as egocentric eye fixations on the target stimuli, compared to their monolingual counterparts. The authors noted that participants’ performance was also associated with increased inhibitory control, with bilinguals outperforming monolinguals on the Simon task of inhibitory control, suggesting that the ability to inhibit prepotent, but egocentric, stimuli may be strongly influenced by domain-general inhibitory control. Similarly, Navarro and Conway (2021) tested bilingual and monolingual participants with the Director task of perspective-taking used by Dumontheil et al. (2010). The researchers found that self-identified bilinguals committed fewer errors when responding to target trials of the director condition (i.e., perspective conflict trials) compared to monolinguals. This finding provided additional support for the influence of bilingualism for tasks that require continuous inhibition of one’s own perspective while managing cognitively demanding secondary tasks.

While previous studies had examined bilingualism within dichotomous group comparisons, Navarro et al. (2022) examined several bilingualism-related traits using an individual differences approach to identify what aspects of the bilingual experience may underlie the effect. The findings indicated that tasks that require inhibition, switching, working memory, and updating processes, such as the simultaneous use of two languages and frequent switching between languages, were significant predictors of ToM performance, whereas tasks that were less cognitively demanding (e.g., knowledge of multiple languages without substantial language use) were not predictive of ToM performance, suggesting that the reason why some bilinguals outperform monolinguals in ToM may be related to the domain-general demands of certain bilingual tasks rather than a general improvement. These results were further supported by Navarro and Rossi (2024) in a study exploring the mediating role of inhibition in the relationship between metalinguistic awareness and ToM. The researchers found that perspective-taking in a sample of linguistically diverse adults was significantly predicted by metalinguistic awareness, but inhibitory control partially mediated this relationship. The results suggested that both cognitive ability and mental-state representations play a role in ToM performance, and that, interestingly, external factors like socioeconomic and demographic variables (e.g., education, culture) reduced the overall effects of both metalinguistic awareness and inhibitory control for perspective-taking, indicating that a multivariate interplay of mechanisms may underlie this effect.

### 2.2. Individual Differences Approaches to Bilingualism and Theory of Mind

Bilinguals use their languages for multiple purposes, across various domains, and with various individuals (Gasser, 2000; Jaccard & Cividin, 2001; Kupisch & Rothman, 2018; Luk & Bialystok, 2013). As such, an increasing number of studies have adopted individual differences or multivariate approaches to studying the relationship between bilingualism and ToM. For example, Tiv et al. (2022) used personal network analysis to explore differences in mentalizing ability among bilinguals who resided in high linguistically diverse areas in Canada and less linguistically diverse areas in the United States. They found that the degree to which the bilinguals’ social-network variables linked unconnected variables (i.e., general betweenness) was a predictor of ToM across participants in both geographical areas. In contrast, the degree to which participants’ networks linked otherwise unconnected language groups (i.e., language betweenness) predicted ToM performance but only in high linguistically diverse areas (i.e., Montreal, Canada), where active bilingualism is deeply rooted and common-place. The results indicated that living in environments that require frequent resolution of language-based perspective conflicts may influence ToM performance as a result of environment-dependent adaptive cognitive strategies (Titone & Tiv, 2023). Other studies that examine variability in bilingualism and ToM have shown that perceived irony was related to better ToM performance, but only in high linguistically diverse areas (Tiv et al., 2023), further emphasizing the influence of environmental and contextual factors.

As growing research has shown, individual differences in bilingualism, including linguistic exposure, engagement, and intention are critical factors to understanding behavioral outcomes related to bilingualism (Titone & Tiv, 2023; Luk & Bialystok, 2013; DeLuca et al., 2019; Navarro & Rossi, 2023; Hartanto & Yang, 2020). For this reason, research with bilinguals should be thoughtful and deliberate when deciding the bilingual criteria, traits, and behaviors that researchers expect may influence performance on other social, cognitive, and linguistic outcomes. While these frameworks highlight the importance of variability, the current study did not take an individual differences approach. Instead, our goal was to isolate a specific profile of functionally engaged bilingualism by using strict inclusion criteria.

In line with these theoretical frameworks, the current study defined “active bilinguals” as individuals who reported moderate-to-high proficiency (≥6 out of 10) in their second language and regular weekly use of both languages across multiple communicative domains such as personal activities, social interactions, and media consumption. We use “L1” to refer to the language participants identified as their dominant or first-acquired language, and “L2” as the second or less-dominant language. This categorical approach allowed us to test whether specific traits associated with active bilingualism—rather than bilingualism in general—would be sufficient to support enhanced ToM performance. These criteria were selected to ensure that included bilinguals were not only proficient but also actively engaged in contexts that demand cognitive flexibility, such as frequent language switching and inhibition of interference. This approach aligns with recent work emphasizing the importance of both language proficiency and real-world usage patterns in shaping the cognitive outcomes of bilingualism (DeLuca et al., 2019; Titone & Tiv, 2023).

### 2.3. Current Study

Consistent with growing research, ToM ability seems to vary across individuals and contexts (Warnell & Redcay, 2019; Cutting & Dunn, 1999) and both experience-based factors (e.g., language competence) and cognitive abilities (e.g., reasoning ability) may work in tandem to affect performance (Navarro et al., 2020; Luk & Bialystok, 2013; DeLuca et al., 2019).

The present study aims to replicate and extend the findings of Navarro and Conway (2021), who reported that bilingual young adults outperformed monolingual peers on the Director Task, particularly on trials requiring perspective taking. Although these findings are promising, they raise important questions about the cognitive mechanisms that underlie the observed effects of bilingualism for ToM. In particular, it is possible that domain-general cognitive abilities—such as fluid intelligence—support ToM performance by enabling individuals to reason flexibly, resolve conflicting perspectives, and inhibit egocentric responses (German & Hehman, 2006; I. A. Apperly et al., 2008). Thus, any benefits of bilingualism may be contingent upon an individual’s broader cognitive ability. To explore these questions, the current study builds on prior work by investigating whether bilingualism influences ToM performance in young adults, and whether fluid reasoning influences this effect.

## 3. Method

### 3.1. Design and Participants

The present study was part of a larger data collection effort including a total of 250 participants from New Mexico State University. The experiment was a 2 (Trial Type: Target, Control) × 2 (Condition: Director, No Director) × 2 (Language Group: Active Bilingual, Monolingual) mixed factorial design with trial type and condition as within-subjects variables and language group as a between-subjects variable. Although participants self-identified as either bilingual or monolingual, final classification was based on objective criteria derived from the Language Experience and Proficiency Questionnaire (LEAP-Q; Marian et al., 2007). Participants were classified as monolingual if they reported exposure to only one language (English) from birth and reported minimal or no use of any second language. In contrast, participants were classified as active bilinguals, if they reported learning or using two languages for most of their lives, scored ≥6/10 in L2 proficiency, and reported regular use of both languages across two or more distinct communicative contexts— such as interacting with others, managing personal tasks, or consuming information and entertainment. Participants who self-identified as bilingual but did not meet the criteria for active bilingualism were excluded from the final sample. Based on these criteria, the final sample included 66^1^ participants who were categorized as either monolingual (n = 39) or active bilingual (n = 27) (see Table 1).

#### Participant Language and Background

The mean age of active bilingual participants in the study was 20 years (SD = 3.09); participants reported acquiring their first language (L1) at a young age (M = 2.47 years, SD = 2.26) and their second language (L2) shortly thereafter (M = 3.67 years, SD = 3.57), suggesting that the majority of the sample were early bilinguals. Regarding language identity, the majority of participants identified English as their L1 (79%), followed by Spanish (16%), and a smaller portion reported another language as their L1 (4%). Conversely, Spanish was most commonly reported as participants’ L2 (72%), followed by English (18%), and other languages (9%). A similar pattern emerged in terms of the language acquired first (A1), with 53% acquiring Spanish first, 38% English first, and 8% acquired another language first. The second language acquired (A2) was Spanish for 32% of participants, English for 5%, and other languages for 17%, suggesting that many participants were exposed to more than two languages or experienced overlapping language development trajectories.

In terms of self-reported language proficiency as shown in Table 2, participants reported relatively high proficiency across all three language domains—comprehension, speaking, and reading—for their L1: comprehension (M = 8.52, SD = 1.93), speaking (M = 8.39, SD = 1.93), and reading (M = 8.41, SD = 1.98); for their L2, proficiency ratings were slightly lower, especially in speaking and reading: comprehension (M = 7.12, SD = 2.32), speaking (M = 6.13, SD = 2.53), and reading (M = 6.31, SD = 2.51). This suggests a relatively balanced but L1-dominant bilingual profile, as it is commonly found in U.S. bilinguals. Daily exposure to each language also reflected a stronger dominance of the L1, with participants reporting spending approximately 73.48% (SD = 19.02) of their daily language use in their L1, and 27.35% (SD = 18.87) in their L2.

### 3.2. Measurements

**Figure 1:**
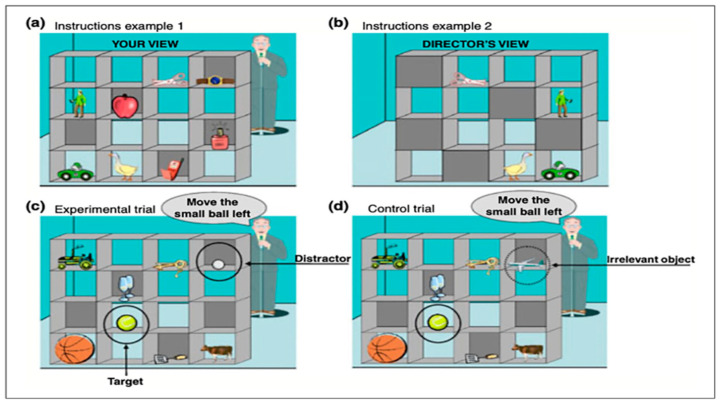
Illustration of the Director task, which is used to assess Theory of Mind (ToM). During the instructions phase, participants are shown an example of their view (**a**) and the corresponding Director’s view (**b**) for a given trial. During the experiment phase, participants may encounter experimental trials (**c**) or control trials (**d**). The task requires the participant to follow the oral instruction given by the Director. In experimental trials (**c**), the participant is required to move the target item (tennis ball) and ignore the distractor item (golf ball) if they take into account the Director’s perspective. In control trials, an irrelevant object is shown instead of the distractor. The figure is a reprint with permission from the original source (Dumontheil et al., 2010).

*Theory of Mind.* The Director task (Figure 1) was used to assess ToM perspective-taking component, which has received much attention in the adult neurotypical population (e.g., Ferguson & Cane, 2017; Pile et al., 2017; Samuel et al., 2019).

Conditions: Participants in the Director condition were instructed to understand the Director’s perspective. They were shown a shelf from the Director’s point of view, and it was made clear that the Director could not see anything in the obscured compartments. This condition assesses theory of mind by demanding the participant to recognize that the Director’s perspective deviates from theirs. Participants are shown the identical shelf in the No-Director condition, but the Director is no longer behind it. Instead, participants are instructed to disregard any items placed in the slots with grey backgrounds. This condition does not necessitate the use of ToM but instead requires the participant to block the overt input while remembering the strategic rule, requiring only general executive control.

Trial Types: In target trials, the shelf displayed a competing object that could be the most appropriate reaction, but only from the participant’s perspective (see Panel (c) in Figure 1). To reply correctly, participants had to consider the Director’s point of view and avoid clicking on the competing item that was only visible to them. In control trials, the target item was always the best answer from both perspectives, and there was no competing object in one of the grey compartments (i.e., Panel (d) in Figure 1). Filler trials are shelf items with no competitors that are visible to both the Director and the participant. The same three types of experimental trials were used in the No Director condition. Target and control trials were never shown in the same stimulus (i.e., shelf display) and were presented in random order throughout the task. Before the initial audio instruction, each stimulus was presented for two seconds, and each stimulus included three sound instructions. Participants responded to a total of 16 trails with eight control trials, eight experimental trials and 48 fillers in each condition. The measure of performance was average accuracy across trials.

*Fluid intelligence (Gf).* To assess fluid intelligence, participants completed two abstract reasoning tasks: Raven’s Progressive Matrices (J. C. Raven, 1938) and the MaRs-IB test (Chierchia et al., 2019). First, in the MaRs-IB, participants were presented with a 3 × 3 matrix, in which 8 cells contain abstract shapes, and one cell is empty. The task required the participant to find the missing shape among four alternatives by analyzing the relationships between the shapes in the matrix based on shape, color, size, and position. In the study, participants were given 40 out of the 80 original trials (odd numbered). Participants were given instructions, an example and five practice items before the task. Second, a short version of Raven’s figural inductive reasoning task was used. Participants were administered odd numbered trials. In this task, each item is part of a pattern of eight black and white figures arranged in a 3 × 3 matrix in which the last bottom right figure is missing. At the bottom of the matrix is a list of eight possible figures to choose from. Participants must select the correct missing figure to complete the pattern. A composite score for accuracy was created by averaging responses on both the Gf measures. We selected these Gf measures specifically to minimize cultural and linguistic bias and directly assess pattern recognition, abstract reasoning, and the inhibition of misleading cues (J.C. Raven, 1938; J. Raven, 2000; P. A. Carpenter et al., 1990). These abilities are theorized to support perspective-taking by enabling individuals to flexibly suppress their own egocentric viewpoint and simulate others’ mental states (I. Apperly, 2010; Qureshi et al., 2010).

*Attention Control:* Attentional control was included in the present study to assess baseline executive functioning across participants, given its established relevance to ToM performance in both children and adults (Carlson & Moses, 2001). Three tasks designed by Burgoyne et al. (2023) were used to assess Attention control (i.e., complex versions of Stroop, Simon, and Flanker). These tasks were constructed to add an additional level of complexity to the traditional conflict paradigms. For instance, the complex Stroop task follows the traditional paradigm where participants are asked to respond based on the color of the text, ignoring the word. Instead, in this version participants must select the response option whose meaning matches the color of the target stimulus. Similarly, for the Simon task, participants were presented with an arrow stimulus, pointing to either right or left, with response options “RIGHT” and “LEFT”. Participants are required to select the response option that corresponds to the direction the arrow is pointing, though the location of the stimulus and the responses were randomly interchanged to add additional complexity. Finally, in Flanker, participants were presented with a target stimulus and response options consisting of five arrows that point in different direction (e.g., > > < > >). Participants must select the response whose central arrow points in the same direction as the flanking arrows in the target stimulus. For all three tasks, participants earn points for correct responses and lose points for incorrect responses. All tasks include a practice phase and test phase. The number of correct responses minus the number of incorrect responses was calculated for each task (For a more through descriptions of these tasks, see Burgoyne et al., 2023). In this study, AC was used to confirm group equivalence between active bilingual and monolingual participants on core executive control abilities. This allowed us to rule out the possibility that observed differences in ToM performance were attributable to broader cognitive differences between groups.

*Bilingualism.* Participants were administered the Language Experience and Proficiency Questionnaire (LEAP-Q; Marian et al., 2007). The questionnaire required the participants to report extensive information about their language experiences throughout their life span, including dominant language, language acquisition order, average time of usage per language, years residing in a country where the language is spoken, and the total number of languages spoken. In this study, we defined “L1” as the language participants identified as either their native or most dominant language, and “L2” as their secondary language. The LEAP-Q was used to classify participants as active bilinguals if they reported using both L1 and L2 weekly across at least two different life contexts and rated their L2 proficiency at 6 or higher on a 10-point scale for both speaking and comprehension. These criteria were selected based on guidelines by Surrain and Luk (2019), who recommend multidimensional assessments of bilingualism that consider both language proficiency and usage across contexts (e.g., social, academic, professional, personal, and recreational contexts).

This classification includes both balanced bilinguals and L2-dominant individuals, as long as they engage regularly with both languages. Participants who reported no use of more than one language (<1%), low to no proficiency in one language domain, or recent to new exposure to a second language with no significant proficiency were excluded from the bilingual group. Our thresholds were informed by prior work showing that only cognitively demanding bilingual behaviors—such as frequent switching and high dual-language engagement—are predictive of ToM outcomes (Navarro et al., 2022; Navarro & Rossi, 2023, 2024). These criteria were selected to ensure that participants were functionally engaged bilinguals who use both languages consistently, reflecting the cognitive demands hypothesized to support Theory of Mind performance. We adopted these thresholds in alignment with previous research using the LEAP-Q and to maintain consistency with the previous Navarro and Conway (2021) study.

### 3.3. Procedure

All tasks were administered in person via Qualtrics and E-prime. Participants were randomly assigned to three counterbalanced orders according to an unbalanced Latin square design. Each order was counterbalanced based on the constructs assessed by the task (i.e., AC, Gf, and ToM), such that Order 1: ToM tasks, AC tasks, Gf task, Order 2: AC tasks, Gf task, ToM tasks, Order 3: AC task, ToM tasks, EF tasks. Participants then completed the LEAP-Q questionnaire as the last item.

For the Director task, all participants were given a standardized set of instructions and practice. In the Director condition, they were told that the director would give them a series of instructions about where to move different objects in the shelf. The participants were explicitly told that the Director did not have the same view as their own perspective and therefore could not see the objects in the obscured slots. They were also asked to think of the cartoon as a real person. In addition, participants were shown a stimulus from the perspective of the Director and were given examples of objects that both them and the Director could see and objects only the participant could see. Before the test trials, the participants were presented with three practice trials where the participant had to move three objects that the Director indicated. All participants were administered the Director condition first and the No Director condition second to avoid participants using the strategy provided in the No Director condition to respond to trials in the Director Condition (Dumontheil et al., 2010).

## 4. Results

Table 3 presents descriptive statistics for accurate responses on the director task and cognitive tasks for bilingual and monolingual participants. For each group, mean task accuracy (M), standard deviation (SD), minimum/maximum scores, skewness, and kurtosis are provided for each trial type in the Director Task (DT) and the No Director Task (NODT), as well as composite scores of Fluid intelligence (Gf) and Attention Control (AC).

In the Director Task, the mean accuracy scores for the bilingual participants on the target condition was comparatively lower (M = 0.45, SD = 0.30) than in the Control condition (M = 0.84, SD = 0.21) suggesting that the target condition was more challenging than the control. A similar pattern was observed for the monolingual group in average accuracy for target trials (M = 0.29, SD = 0.27) compared to control trials (M = 0.84, SD = 0.14). Skewness and kurtosis values indicated an acceptable to moderate deviations from normality, especially in the Control condition for active bilinguals (Skew = 2.22, K = 5.99). In the No Director Task, both groups showed reduced accuracy in the Target condition relative to Control, though the differences were less pronounced than in the DT. Active bilinguals scored M = 0.71 (SD = 0.30) in the Target condition and M = 0.96 (SD = 0.13) in the Control condition while Monolinguals scored M = 0.70 (SD = 0.34) and M = 0.99 (SD = 0.07), respectively. For the cognitive ability measures, both fluid intelligence (Gf) and attention control (AC) scores were nearly identical between active bilinguals and the group difference was not statistically significant (both *p* > 0.05). These results indicated that the two language groups were matched on general cognitive abilities, minimizing the possibility that differences in ToM performance (e.g., on the Director Task) were related to disparities in domain-general abilities.

## 5. Main Analysis

### 5.1. ToM and Bilingualism

A 2 (Trial Type: Control, Target) × 2 (Condition: Director, No Director) × 2 (Active Bilingualism Group: Yes, No) mixed factorial ANOVA was conducted on Director task accuracy. As observed in previous studies, there was a significant main effect of Condition, *F*(1, 64) = 99.83, *p* < 0.001, η^2^_p_ = 0.609, indicating that participants performed significantly worse in the Director condition compared to the No Director condition. A significant main effect of Trial Type was also observed, *F*(1, 64) = 136.02, *p* < 0.001, η^2^_p_ = 0.680, such that participants were more accurate on Control trials than Target trials, consistent with the increased demands of perspective-taking required in the Target trials.

**Figure 2:**
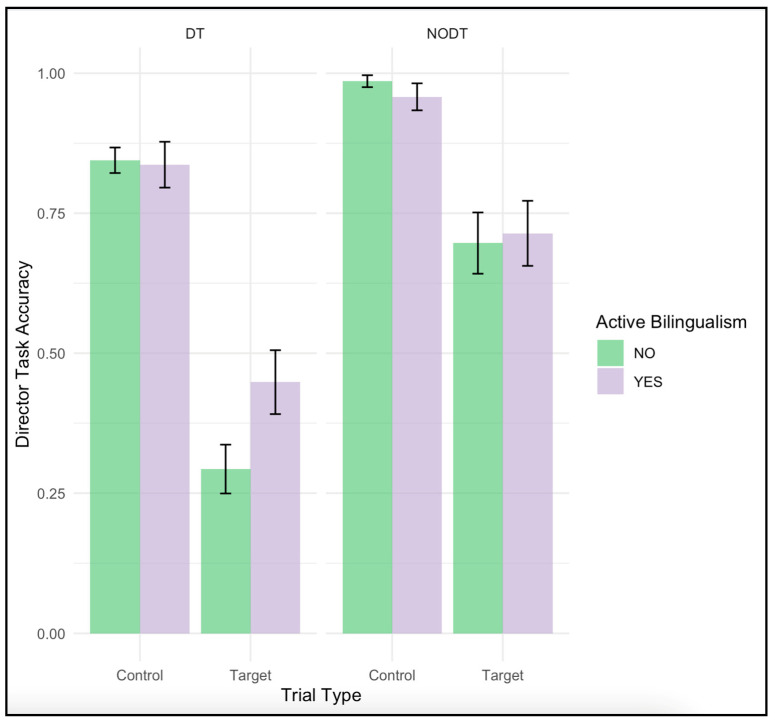
Accuracy by Bilingualism and trail type per condition.

The three-way interaction between Trial Type, Condition, and Active Bilingualism presented a medium not significant effect, *F*(1, 64) = 1.48, *p* = 0.228, η^2^_p_ = 0.023. While the three-way interaction was not significant, Figure 2 illustrated a trend similar to that reported by Navarro and Conway (2021). Specifically, bilingual participants appeared to outperform monolinguals on Target trials within the Director condition—the condition requiring the greatest perspective-taking ability. While not statistically significant, this pattern was theoretically consistent with the proposal that bilinguals may be better able to inhibit egocentric responses in demanding social-cognitive tasks, highlighting the importance of exploring individual difference factors that may moderate Theory of Mind (ToM) performance under cognitively demanding conditions. To further explore whether this effect may be affected by the limited sample size, a Welch’s independent-samples *t*-test was conducted on target trials of the director task condition comparing active bilinguals and monolinguals. The analysis showed a significant medium-size effect, *t*(52.88) = −2.16, *p* = 0.035, *d* = −0.55, where active bilinguals were more accurate than than monolinguals (bilinguals: M = 0.45, SD = 0.30; monolinguals: 0.29, SD = 0.27). These results suggest that a larger sample size may be needed to identify the small to medium effect of bilingualism for ToM.

### 5.2. ToM, Bilingualism and Gf

To further build on the previous results, fluid intelligence (Gf) was included in the model as a covariate to examine whether individual differences in reasoning ability account for variability in bilingual Theory of Mind performance. This model retained the original 2 (Trial Type: Target, Control) × 2 (Condition: Director, No Director) × 2 (Active Bilingualism: Yes, No) mixed factorial structure.

**Figure 3:**
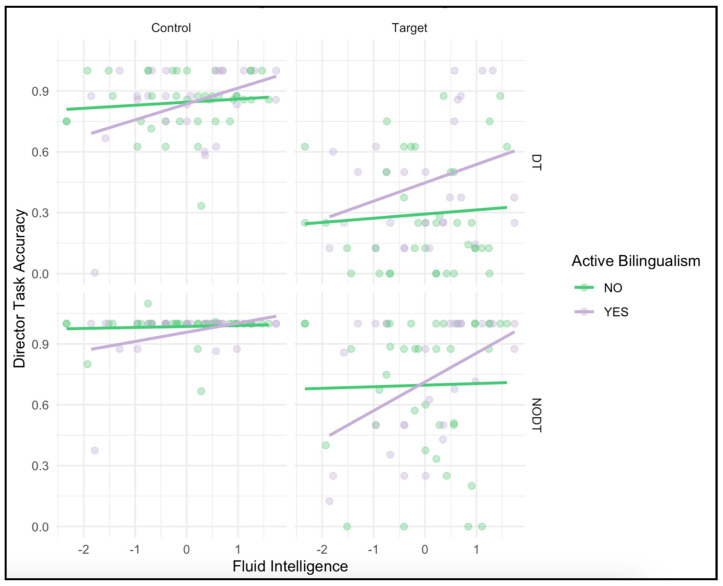
Interaction between Gf and Bilingualism across trial types and conditions.

The model revealed a significant main effect of fluid intelligence, *F*(1, 62) = 8.24, *p* = 0.006, η^2^_p_ = 0.012, suggesting that participants with higher reasoning ability performed more accurately overall. In addition, the analysis revealed a significant interaction between bilingualism and Gf, *F*(1, 62) = 4.76, *p* = 0.033, η^2^_p_ = 0.071, suggesting that fluid intelligence moderated the relationship between bilingualism and perspective-taking performance. Specifically, active bilinguals with higher Gf were more accurate in trials that required taking the perspective of the director (i.e., target trials) compared to bilinguals with lower Gf and to monolinguals, indicating a possible moderating role of cognitive ability in the bilingual ToM effect (Figure 3). Importantly, the main effect of Trial Type, *F*(1, 62) = 134.57, *p* < 0.001, η^2^_p_ = 0.685, and Condition, *F*(1, 62) = 96.96, *p* < 0.001, η^2^_p_ = 0.609, remained significant in this model. The Trial Type × Condition interaction also remained robust, *F*(1, 62) = 17.41, *p* < 0.001, η^2^_p_ = 0.219, consistent with the idea that performance was especially challenging on target trials in the Director condition. The three-way interaction among Trial Type, Condition, and Bilingualism remained non-significant, *F*(1, 62) = 1.48, *p* = 0.23, η^2^_p_ = 0.023, suggesting that group-based differences in accuracy did not vary across conditions even after accounting for Gf. Overall, the results suggest that Gf plays a meaningful role in task performance, and that improved ToM performance attributed to bilingualism may be better understood in relation to underlying reasoning abilities.^2^

## 6. Discussion

The present study aimed to replicate and extend previous findings by examining how bilingualism may influence Theory of Mind (ToM) performance in adults. Consistent with prior work, participants exhibited significantly lower accuracy on target trials compared to control trials, and accuracy was significantly reduced in the Director condition relative to the No Director condition, demonstrating the increased cognitive demands of perspective-taking tasks (Navarro et al., 2020) and providing additional support for the view that adults’ ToM is subject to individual differences (Navarro & Conway, 2021). Although we did not observe a statistically significant three-way interaction between Trial Type, Condition, and Language Group, the overall pattern of results and subsequent *t*-test showed support for the effect of bilingualism for ToM. These findings are discussed below.

In the current study, we included a highly selective set of criteria for inclusion in the bilingualism group (i.e., active bilinguals); active bilinguals showed higher performance on perspective-dependent target trials of the Director Task when compared to monolinguals. This suggests that there is a small effect of bilingualism for perspective-taking ToM, which may have required additional statistical power to identify (which was confirmed by *posthoc* power analyses). However, it also emphasizes the challenges of identifying effects of bilingualism on behavioral outcomes, and their relative real-world applications. Overall, the results support prior research suggesting that bilinguals may exhibit enhanced ToM under cognitive load (Rubio-Fernandez & Glucksberg, 2012; Goetz, 2003) but also underscores the variability in these effects across bilingual profiles and tasks.

The study also presented novel evidence of an interaction between fluid intelligence and bilingualism for ToM, suggesting that performance in the target trials of the director task increased as a function of fluid intelligence, which had only been observed among older adults (German & Hehman, 2006; Goring & Navarro, in press). This finding expands previous research on ToM performance among bilinguals, suggesting that while bilingualism may lead to small gains in perspective-taking ToM, this effect may be dependent on a bilingual’s ability to reason about novel tasks (German & Hehman, 2006; I. A. Apperly & Butterfill, 2009). Given that bilingual participants were not significantly different from monolinguals in either fluid intelligence or attention control, this interaction supports previous research suggesting a strong influence of domain-general abilities on social cognitive performance under cognitively taxing circumstances (Navarro & Rossi, 2024; Navarro, 2022; German & Hehman, 2006), among otherwise healthy adults.

In particular, the findings of this study suggest that engaging in active bilingual practices may, at higher levels of ability, facilitate the recruitment of compensatory processes that reduce cognitive demands during task completion. This view would imply that individuals with higher levels of cognitive abilities benefit the most from bilingual-related experience (Hambrick & Engle, 2003) by possibly engaging in greater mental state representation or abstract reasoning (Sperber, 2000; M. Carpenter et al., 2002). This finding further raises important questions about whether this effect is unique to Gf or generalizes to other domain-general abilities. While Gf captures core reasoning and inhibition skills, it overlaps conceptually with working memory and attention control—both of which have been implicated in ToM performance in past work (I. Apperly, 2010; Qureshi et al., 2010; Navarro et al., 2020). Future studies should examine whether similar moderating patterns emerge for other cognitive abilities, or whether Gf represents a distinct executive mechanism in the bilingual-ToM link.

A key consideration in interpreting these results lies in the variability of bilingualism itself. As recent work has emphasized (e.g., Paap et al., 2025; Feng et al., 2023), bilingualism is not a binary trait, but a highly individualized experience shaped by age of acquisition, proficiency, frequency of use, language context, and dominance. Studies that rely solely on self-reported bilingual status may inadvertently over-represent unbalanced bilinguals or late L2 learners. Sample heterogeneity may contribute to inconsistencies across studies examining bilingualism correlates and highlights the need to move beyond categorical comparisons and towards an individual differences approach that aligns bilingual experience profiles with the specific domain-general processes hypothesized to underlie complex higher-order cognitive abilities, such as ToM.

Along these lines, not all bilingual traits are functionally equivalent. While early bilinguals with frequent language switching may show advantages in ToM or executive control tasks, occasional L2 learners with limited exposure may not. Matching specific bilingual experiences to the cognitive processes they are hypothesized to affect is important. The current study established rigid criteria to include bilingual participants whose language experiences aligned with the ToM-related processes that have been identified in previous research (Navarro et al., 2022), namely frequent and enduring language selection, inhibition, and use based on context, interlocutor, or goals and intentions. Future work should consider more targeted recruitment and developing continuous bilingualism measures to properly assess samples of interest.

Furthermore, culture has been found to be associated with differences in ToM performance, for example, Wu and Keysar (2007), found that interdependent individuals outperformed their independent counterparts in a perspective-taking ToM task. While the present study included bilingual participants from diverse linguistic and cultural backgrounds, we took steps to assess whether cultural orientation may have influenced performance. Specifically, we administered a standardized measure of cultural values (the Self-construal Scale, Kitayama et al., 2014) assessing both independence and interdependence, which are core dimensions of individualist and collectivist orientations (Singelis, 1994). Results indicated no significant group differences between active bilinguals and monolinguals in either independence (Active bilingual = 78.48, Monolingual = 78.13, *p* = 0.90) or interdependence scores (Active bilingual = 69.67, Monolingual = 68.74, *p* = 0.73). These findings suggest that cultural value orientation was not a likely confounding factor in this sample, although future work may benefit from further exploring the intersection of language experience and culturally shaped mentalizing styles (Wu & Keysar, 2007).

From a measurement perspective, the task used to assess ToM also deserves further scrutiny. While the Director Task is a widely used and ecologically valid measure of adult perspective-taking, it is not without limitations. Prior work has suggested that performance on the Director Task may conflate Theory of Mind with domain-general executive control processes (Quesque & Rossetti, 2020; Rubio-Fernandez, 2017). However, in the present study, we found no significant group differences in either fluid intelligence (Gf) or attentional control (AC), which suggests that any observed differences on the ToM task are unlikely to be driven by broader executive function. Although the inclusion of a No-Director control condition in our design helps isolate perspective-taking demands, the study’s reliance on a single ToM domain limits our ability to draw conclusions about broader mentalizing abilities. It is worth noting that the Director Task specifically targets the perspective-taking component of ToM (Level 1 mentalizing; see Flavell et al., 1981), a subdomain particularly relevant to social coordination, language use, and on-line communication. Our interest in perspective-taking is grounded in previous findings that suggest bilingualism may support this specific subcomponent of ToM due to increased demands on inhibition and switching during language use (Navarro & Conway, 2021; Navarro et al., 2022).

It is also important to acknowledge that convergent validity across ToM tasks is limited by the fact that most measures of ToM are strongly process-impure and tap into different subcomponents of mentalizing (Navarro, 2022). While some studies suggest stronger convergence between the Director Task and tasks like the Reading the Mind in the Eyes Test (RMET), others (e.g., irony comprehension or faux pas recognition) engage different mechanisms, including socio-emotional and affective inference, which may not be directly comparable to the perspective-taking demands targeted here. As such, there is limited evidence that other ToM components would assess the same cognitive processes engaged during active bilingualism.

Furthermore, highlighted by recent work (Feng et al., 2023; Navarro, 2022), there is considerable inconsistencies in how ToM is operationalized across studies, ranging from traditional false-belief tasks to interactive communication-based paradigms (Warnell & Redcay, 2019; Quesque & Rossetti, 2020). The differences in task paradigm, demands, and ToM subcomponents make cross-study comparisons difficult and may explain why some studies have failed to find bilingualism effects while others report robust effects. Given our study’s specific focus on visual perspective-taking and knowledge attribution, the Director Task remains well-suited to test the hypothesis that active bilinguals show advantages due to frequent practice in tracking others’ mental states during real-world communication. Continued efforts to standardize and validate ToM measures, particularly those used in bilingual contexts, will be crucial in clarifying whether and how bilingual experience modulates social-cognitive reasoning.

Finally, we acknowledge several limitations. Although our results replicated the overall experimental effects found by Navarro and Conway (2021), as mentioned, our stricter inclusion criteria—particularly for identifying active bilinguals—and technical issues during data collection reduced our final sample size and likely limited our power to detect subtle interaction effects. Nonetheless, a planned comparison between bilinguals and monolinguals on target trials of the Director Task revealed a statistically significant difference with a medium effect size (d ≈ 0.55). This suggests a meaningful group-level difference that aligns with prior work (e.g., Rubio-Fernandez & Glucksberg, 2012; Goetz, 2003), though such effects may be moderated by domain-general abilities and contextual factors.^3^

Additionally, our reliance on self-reported language experience, though common in the literature, may have introduced classification noise. While all bilingual participants met the minimum inclusion threshold for active L2 use and proficiency (≥6/10), there was notable variability in their reported L2 skills (e.g., speaking M = 6.13, SD = 2.53), which may have diluted group-level effects. To contextualize this further, we compared self-reported L1 proficiency across groups and found that monolinguals reported consistently higher L1 proficiency than bilinguals.^4^ These findings are consistent with prior research showing that language self-assessments can vary based on context (e.g., rating L1 in isolation vs. alongside L2), and may reflect known limitations of self-report-based language measures (Gollan et al., 2012; Bice & Kroll, 2019; Olson, 2024). Future studies would benefit from using objective measures of language proficiency to reduce measurement error and better capture functional bilingualism.

Overall, while our findings do not conclusively support a main effect of bilingualism on adult ToM, they provide evidence of a subtle interplay between key bilingualism-related traits and cognitive abilities that add to the ongoing debate about the processes involved in social cognition, and reinforce the importance of controlling for task complexity, measurement precision, and clear conceptualizations of bilingualism to better understand how language experience shapes social cognition.

## Figures and Tables

**Table 1:** Language use and proficiency of Active Bilinguals.

**Varibales**	***M***	***SD***
Age	20	3.09
L1 AoA	2.47	2.26
L2 AoA	3.67	3.57
**Self-reported L1 and L2 frequency**		
	**L1**	**L2**
English	79%	18%
Spanish	16%	72%
Other	4%	9%
**Self-reported Language Acquisition Pattern**		
	**Acquired First (A1)**	**Acquired Second (A2)**
English	38%	5%
Spanish	53%	32%
Other	8%	17%

Note. AoA = Age of Acquisition; L1 = Self-identified first language; L2 = Self-identified second language; A1 = Language acquired first; A2 = Language acquired second. Percentages may not total 100% due to rounding. All values are based on self-reports from participants classified as active bilinguals.

**Table 2:** Self-reported language proficiency and daily use.

Skill	L1		L2	
	M	SD	M	SD
Comprehension	8.52	1.93	7.12	2.32
Speaking	8.39	1.93	6.13	2.53
Reading	8.41	1.98	6.31	2.51
Daily Exposure	73.48	19.02	27.35	18.87

Note. Proficiency scores range from 0 (no proficiency) to 10 (fully fluent). Daily exposure refers to participants’ estimated percentage of time spent using each language in a typical day.

**Table 3:** Descriptive statistics for task accuracy and cognitive scores by language group.

Groups	Bilingual Group					Monolingual Group				
	n = 27					n = 39				
	M	SD	Min/Max	Skew	K	M	SD	Min/Max	Skew	K
**Director Condition**										
Target Trials	0.45	0.3	0.12/1	0.63	−0.98	0.29	0.27	0/0.88	0.67	−0.87
Control Trials	0.84	0.21	0/1	2.22	5.99	0.84	0.14	0.33/1	−1.18	2.17
**No Director Condition**										
Target Trials	0.71	0.3	0.12/1	−0.47	−1.36	0.7	0.34	0/1	−0.81	−0.72
Control Trials	0.96	0.13	0.38/1	−3.75	14.4	0.99	0.07	0.67/1.1	−3.36	12.45
**Cognitive Measures**										
Fluid intelligence (Gf)	0.44	0.07	0.30/0.56	−0.18	−0.95	0.43	0.07	0.27/0.55	−0.57	−0.38
Attention Control (AC)	0.97	0.4	0.32/1.70	0.34	−1.07	0.87	0.33	0.20/1.46	−0.14	−1.04

Note. All accuracy scores reflect proportion correct (range: 0 to 1). Skew = Skewness; K = Kurtosis.

## Data Availability

The data supporting the conclusions of this article will be made available by the authors on request.

## Notes

1 Although the total sample included 250 participants, only 88 were able to complete the Director Task due to technological errors. Of those, 66 participants met inclusion criteria for the bilingualism analysis based on language background data and proficiency cutoffs.

2 Reaction times were also analyzed; as expected, there were no meaningful differences in performance across groups or conditions, replicating previous findings.

3 A post-hoc power analysis using G*Power estimated the analysis possessed 67% power to detect a small-to-medium three-way interaction (f = 0.15) in a 2 × 2 mixed factorial design. This suggests that the non-significant interaction observed in our results may reflect a Type II error due to limited statistical power.

4 Monolinguals reported higher L1 proficiency than bilinguals across speaking (*t*(64) = 2.85, *p* = 0.006), comprehension (*t*(64) = 2.61, *p* = 0.012), and reading (*t*(64) = 1.98, *p* = 0.053).
